# Optimization of collimator angle combined island blocking with parked gap achieves superior normal tissue sparing in SBRT planning of multiple liver lesions

**DOI:** 10.1002/acm2.14267

**Published:** 2024-01-23

**Authors:** Maidina Abuduxiku, Xiaoqiang Chen, Shu Zhang, Jiangping Yang, Wenjun Liao, Jianghong Xiao

**Affiliations:** ^1^ Radiotherapy Physics & Technology Center Cancer Center West China Hospital Sichuan University Chengdu Sichuan China; ^2^ Head and Neck Oncology Department Cancer Center West China Hospital Sichuan University Chengdu Sichuan China; ^3^ Department of Radiation Oncology Cancer Center West China Hospital Sichuan University Chengdu Sichuan China; ^4^ Department of Radiation Oncology Affiliated Cancer Hospital of University of Electronic Science and Technology of China Chengdu China

**Keywords:** collimator angle optimization, island blocking, multiple lesions, parked gap, stereotactic body radiation therapy

## Abstract

**Purpose:**

To propose an efficient collimator angle optimization method by combining island blocking (IB) and parked gap (PG) problem to reduce the radiotherapy dose for normal tissue. The reduction will be done with single‐isocenter multi‐lesion volumetric modulated arc therapy (VMAT) for the stereotactic body radiation therapy (SBRT) of liver cancer.

**Methods:**

A novel collimator angle optimization algorithm was developed based on the two‐dimensional projection of targets on a beam's eye view (BEV) plane as a function of gantry and collimator angle. This optimization algorithm minimized the sum of the combined IB and PG (IB & PG) areas from all gantry angles for each arc. For comparison, two SBRT plans were respectively generated for each of the 20 retrospective liver cancer cases with multiple lesions. One plan was optimized using the IB & PG algorithm, and the other plan was optimized with a previously reported optimization algorithm that only considered the IB area. Plans were then evaluated and compared using typical dosimetric metrics.

**Results:**

With the comparable target coverage, IB & PG plans had significantly lower *D_500cc_*, *D_700cc_*, mean dose (*D_mean_*), and *V_15_* of normal liver tissues when compared to IB plans. The median percent reductions were 3.32% to 5.36%. The *D_1cc_*, *D_5cc_*, and *D_mean_* for duodenum and small intestine in IB & PG plans were significantly reduced in a range from 7.60% up to 16.03%. Similarly, the median integral dose was reduced by 3.73%. Furthermore, the percentage of normal liver *D_mean_* sparing when IB & PG plans compared to IB plans, was found to be positively correlated (*ρ* = 0.669, *P* = 0.001) with the inter‐target distance.

**Conclusion:**

The proposed IB & PG algorithm has been demonstrated to outperform the IB algorithm in almost all normal tissue sparing, and the magnitude of liver sparing was positively correlated with inter‐target distance.

## 1 INTRODUCTION

Stereotactic body radiation therapy (SBRT) is a novel locoregional treatment modality that delivers high, ablative doses of radiation to extracranial targets with high precision in a single or few fractions.^1^ Recently, SBRT has been proven to be highly effective with better local control in patients with hepatic malignancies, and it became an important noninvasive alternative for primary and metastatic liver cancer.^2^ However, radiation‐induced liver disease (RILD) and gastrointestinal toxicity are major obstacles to dose escalation in liver SBRT.^2^ Toxicities in normal tissue are generally proportional to the dose absorbed by organs at risk (OARs). Therefore, it is essential to reduce the dose to normal liver and other critical structures, especially when the SBRT of multiple liver lesions is performed.

**FIGURE 1:**
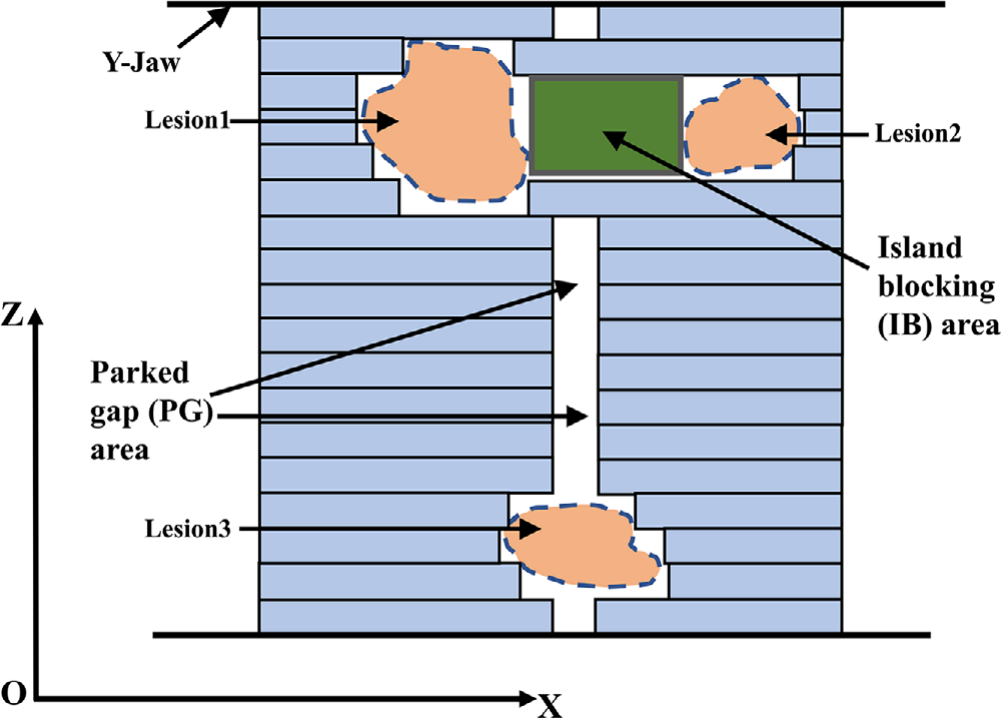
Schematic beam's eye view (BEV) illustration of MLC shaping three lesions. The unshielded normal tissue area of the island blocking (IB) and the parked gap (PG) is exposed to radiation.

Volumetric modulated arc therapy (VMAT) has been established as an advanced treatment technique for delivering SBRT. It offers a better dose distribution and more precise targeting with a much‐reduced delivery time. When compared to conventional multi‐isocenter treatment, some studies have shown that single‐isocenter multi‐lesion VMAT can achieve equivalent plan quality, better clinical efficiency, and potentially less machine scatter and leakage to the patient with lower total monitor units (MUs). It also has greater utility and cost‐effectiveness.^3^, ^4^, ^5^ However, irradiation of multi‐lesion with single‐isocenter brings several problems when multi‐leaf collimator (MLC) delivery is concerned. In the present study, we focused on two issues that would increase unnecessary exposure to surrounding normal structures. One is the island blocking (IB) problem (Figure 1), which occurs when two or more lesions share the same MLC leaf pair. This results in an area of non‐target normal tissue between lesions that is not blocked by MLC. This problem mostly can be avoided by selecting the optimal collimator angles. Several studies^6^, ^7^ have developed optimization algorithms to address the IB problem in the radiotherapy of multiple brain metastases and showed that plans with optimized collimator and couch angles are superior to conventional non‐optimized plans. Other similar studies proposed various kinds of collimator angle optimization methods. One method included a dynamic collimator trajectory optimization algorithm to investigate the effects of collimator angles on plan quality. They have reached a consensus that the optimized collimator angle reduced the dose to normal tissues.^8^, ^9^, ^10^, ^11^, ^12^, ^13^ The other is the parked gap (PG) problem (Figure 1). For example, in some Elekta MLC systems, the opposing MLC leaf pair must maintain a minimum gap of a certain width to avoid possible collisions.^14^ If a leaf pair is needed for shaping the aperture at one control point and yet is not needed at the next control point, this leaf pair should ideally be parked underneath the jaws to avoid unwanted doses to the patient. However, considering the along‐the‐leaf field size and the leaf speed limitation, the closed leaf pairs are parked centrally with gaps.^15^ Webb^15^ especially addressed the problem of parking leaf gaps and investigated the PG problem in the context of single‐lesion irradiation with the fixed jaw Elekta VMAT technique. He stated that collimator rotation was advantageous for reducing the PG area up to 40%. Furthermore, the PG problem becomes especially prominent in single‐isocenter multi‐lesion jaw‐tracking VMAT because it occurs between the lesions and may lead to the formation of high‐dose bridges.

However, most of the previous studies^6^, ^7^, ^8^, ^9^, ^10^, ^11^, ^16^, ^17^ regarding collimator angle optimization only considered the IB problem and neglected the PG problem. Therefore, in this study, we developed a new collimator angle optimization algorithm that combined the IB and PG areas to fully represent the unnecessary open space area of MLC. To our knowledge, for single‐isocenter multi‐lesion SBRT plans, this is the first time that the dosimetric effects of the PG area and the IB & PG area were studied. Furthermore, 20 multi‐lesion liver cancer cases were studied to generate SBRT plans with the IB & PG algorithm. These were compared to plans generated with a conventional algorithm that minimized the IB area alone.

## 2 METHODS

### 2.1 Optimization algorithm description

We computed the IB area based on the approach of previous research^6^, ^7^ and further developed an approach for calculating the IB & PG area over the gantry arc. At first, we defined the patient CT image XYZ coordinate system. The X‐axis direction was from the patient's right to left. The Y‐axis was from the posterior to the anterior direction, and the Z‐axis was from the superior to the inferior direction. The patient's position for this system was head in first supine position. Secondly, the original coordinates, *P(original)*, of the contours of each lesion were obtained from the Raystation scripting program. Thirdly, the gantry spatial rotation of the *g* angle and the collimator spatial rotation of the *c* angle were mathematically expressed as two transformation matrices (*R*(*gantry*) and *R*(*collimator*) respectively). The rotation of the gantry and collimator established a new coordinate system where the new coordinates of the lesions, *P*(*new*), were expressed by the following equation:

$$\begin{array}{c} P\left(new\right)=R\left(collimator\right)\cdot R\left(gantry\right)\cdot P\left(original\right)\\ \;=\left(\begin{array}{ccc} \cos g & -\sin g & 0\\ \sin g & \cos g & 0\\ 0 & 0 & 1 \end{array}\right)\cdot \left(\begin{array}{ccc} \cos c & 0 & -\sin c\\ 0 & 1 & 0\\ \sin c & 0 & \cos c \end{array}\right)\cdot \left(\begin{array}{c} x_{0}\\ y_{0}\\ z_{0} \end{array}\right)\\ \;=\left(\begin{array}{ccc} \cos c\cos g & -\cos c\sin g & -\sin c\\ \sin g & \cos g & 0\\ \sin c\cos g & -\sin c\sin g & \cos c \end{array}\right)\cdot \left(\begin{array}{c} x_{0}\\ y_{0}\\ z_{0} \end{array}\right) \end{array}$$

**FIGURE 2:**
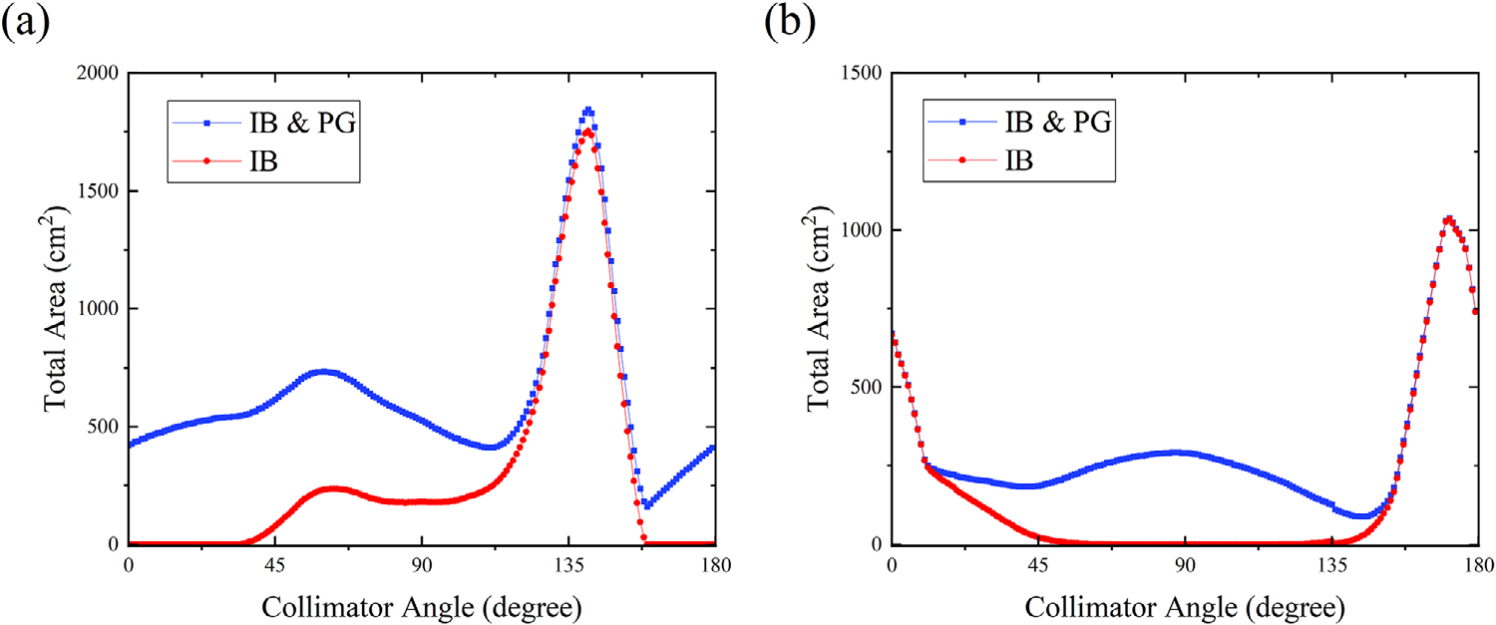
Graphs showing the total IB area and the total IB & PG area for each collimator angle from 0° to 179° with 1° interval for two different cases. (a) An example of a two‐lesion case. The IB area is zero when the collimator angle rotates to 0°−32° and 160°−180°, so equivalently spaced collimator angles of 10°, 20°, 170°, and 180° were selected for four half arcs of the IB plan, and collimator angles of 158°, 159°, 160°, and 161° were selected for the IB & PG plan in this case. (b) An example of a three‐lesion case. Since the IB area is zero when the collimator angle rotates to 72°−113°, so equivalently spaced collimator angles of 80°, 88°, 96°, and 104° were selected for the IB plan, and collimator angles of 143°, 144°, 145°, and 146° were selected for the IB & PG plan. IB, island blocking; PG, parked gap.

In the new coordinate system, the X‐axis represented the MLC along‐the‐leaf motion direction. The Z‐axis represented where the straight line intersects the isocenter and was oriented perpendicular to the MLC motion direction. To describe the spatial geometric information of the targets in relation to the rotation of the gantry and collimator, we used the projected contour coordinates of each target in the two‐dimensional beam's eye view (BEV) plane to calculate the IB area and the IB & PG area. As seen in Figure 1, for a given combination of gantry and collimator angle where there was an area of overlap in the Z‐direction and a separation in the X‐direction between a pair of lesions, a rectangular shape of the IB area was generated. If there was more than one IB region, the union of all the IB area was calculated, and the overlap area of each lesion and IB region was subtracted from the total IB area. Next, the total IB area for each collimator angle was calculated by iteratively summing the IB region at each gantry angle over the gantry arc with a 1° increment. The collimator angle ranged from 0° to 179° with a 1° interval. Since we arranged four half arcs (181° to 0°) for each SBRT plan, the four different collimator angles with the least total IB area were selected as optimized angles for the IB plan. In many cases, especially in the case with two lesions, the optimization had a chance to come up with more than four solutions that completely avoided the IB problem. We kept the collimator angles equivalently spaced to represent all the optimized angles, as shown in Figure 2.

The PG area occurred when there was no projection of lesions in the Z‐direction in the field (Figure 1). The PG area was calculated as the sum of all line lengths of non‐lesion‐projection segments in the Z‐direction in the field over the gantry arc and then multiplied by the width of the minimum leaf gap. For the accelerator used in this study (Elekta Versa HD), the minimum gap between opposing MLC leaves was set to 0.6 cm. Similar to the IB plan approach, the total IB & PG area for each collimator angle was calculated by iteratively adding the summation of the IB area to the summation of the PG area. This was done at each gantry angle over the gantry arc with a 1° gantry angle spacing. Then, the four different collimator angles with the least total IB & PG area were selected as optimized angles for the IB & PG plan.

### 2.2 Patient characteristics and treatment planning

This retrospective study included 20 patients with multiple liver lesions previously treated with SBRT at West China Hospital from 2017 to 2022. The study protocol was approved by the Clinical Research Committee of the study institute. The Institutional Review Board waived the need for informed patient consent. Table 1 summarizes the characteristics of the patients. Each patient underwent the contrast‐enhanced 4D‐CT simulation (SOMATOM Definition AS+, SIEMENS, 120 kVp, 90 mAs). The patient was immobilized using the stereotactic body frame and in the supine position. VMAT plans were generated with the RayStation treatment planning system (RaySearch Laboratories, v9.0) using a collapsed cone algorithm with a grid size of 2 mm. It was customized to the Elekta Versa HD (Elekta Oncology, UK) accelerator, which was equipped with 160 leaves and a 5 mm central leaf width high‐definition MLC system. A 6 MV flattening‐filter‐free (FFF) beam was utilized for all plans.

**TABLE 1:** The characteristics of patients.

Characteristics	No. (%)
Gender	
Male	16 (80%)
Female	4 (20%)
Age (years)	
Median (range)	58 (38–73)
Histology	
Primary	6 (30%)
Metastatic	14 (70%)
Number of lesions	
2	8 (40%)
3	12 (60%)
PTV volume (cm^3^)	
Median (range)	43.14 (14.48–305.62)

Abbreviation: PTV, planning target volume.

An automatic SBRT planning program for liver SBRT was developed in‐house based on previous work.^18^, ^19^ This was used to simulate the manual plan designing process, which included creating the planning auxiliary structures, adding plan objectives and constraints, optimizing, and adjusting parameters during the whole optimization process. The minimum precision level of automatic adjustment (2 cGy) was much more precise than that of manual adjustment (often more than 50 cGy). It has been demonstrated that the automatic SBRT planning program has great repeatability and reproducibility. Additionally, it has the potential to make planning results more objective and stable. In this study, the same initial objectives, constraints, and optimization priorities were used for the IB plan and IB & PG plan for a given case, and the initial objectives and constraints were set according to the prescription. These optimization parameters were adjusted multiple times in the process of optimization to produce a quality plan. The parameter adjustments were based on each optimized objective value to ensure that the objective value was in the range of 0.001 to 0.003 (10 to 30 times the 0.0001 tolerance).

The prescription dose to the planning target volume (PTV) was 50 Gy in five fractions for all patients. All plans were normalized to a prescription dose at 95% of PTV volume. The isocenter was located at the geometric center of all targets. The jaw‐tracking function was utilized, and both plans used the same gantry arcs for each patient. Thus, only the planning parameters of the collimator angles were changed between the initial conditions of IB plan and the IB & PG plan.

### 2.3 Plan evaluation

The parameters used to assess plan quality included the homogeneity index (*HI*), the conformity index (*CI*), the gradient index (*GI*), the *MUs*, the integral dose (*ID*), and the dose indices of the PTV and OARs. The *HI* was defined as *HI* = (*D_2%_*–*D_98%_*)/*D_50%_*, where *D_V_* was the absorbed dose that covers a specified volume.^20^ The *CI* was defined according to the Paddick Index equation. *CI* = (*TV_PIV_* × *TV_PIV_*)/(*TV* × *PIV*), where *TV_PIV_*, *TV*, and *PIV* represent the volume of PTV covered by the prescription isodose volume, the targets volume, and the prescription isodose volume, respectively. *GI* was defined as *V_50%_*/*V_100%_*, where *V_50%_* and *V_100%_* are the volumes receiving 50% and 100% of the prescription dose. *ID* was calculated as *D_meanBody_* × *V_Body_* − *D_meanPTV_* × *V_PTV_*, where *D_meanBody_*, *D_meanPTV_*, *V_Body_*, and *V_PTV_* represent the mean dose of all voxels defined as body, the mean dose of PTV, the body volume, and the PTV volume, respectively.^21^ We followed the normal tissue dose constraints suggested by Timmerman^22^, ^23^ and the RTOG‐1112 protocol^21^ for SBRT in five fractions. The constraints of targets and critical OARs included: PTV, *D_95%_* = 50 Gy, *D_max_* (maximum dose) < 75 Gy (150% of the PTV prescription dose); Liver, *V_D_*_< 19.2 Gy_ > 700 cc (at least 700 cc of the normal liver volume receiving < 19.2 Gy) or *D_mean_* (mean dose) < 13 Gy; Duodenum and stomach and small intestine, *D_1cc_* < 30 Gy or *D_5cc_* < 26.5 Gy. Wilcoxon signed ranks test (SPSS, version 26; USA) was applied to analyze the differences between the plans. Spearman's rank correlation was used to analyze the correlation between two variables. And *P* < 0.05 (2‐tailed) was considered statistically significant.

## 3 RESULTS

Figure 2 shows the illustrative examples of the total IB area and the total IB & PG area over the gantry arc for all collimator angles from 0° to 179° with 1° interval for one two‐lesion case (Figure 2a) and one three‐lesion case (Figure 2b). It can be observed from these figures that there are quite large differences between the total IB area and the total IB & PG area for most collimator angles. The collimator angles with minimum total IB area differ significantly from the collimator angles with minimum IB & PG area. The difference between the total IB & PG area and the total IB area for each collimator angle represents the total PG area. It can be seen from Figure 2 that the PG area usually becomes larger when the IB area is relatively small.

**FIGURE 3:**
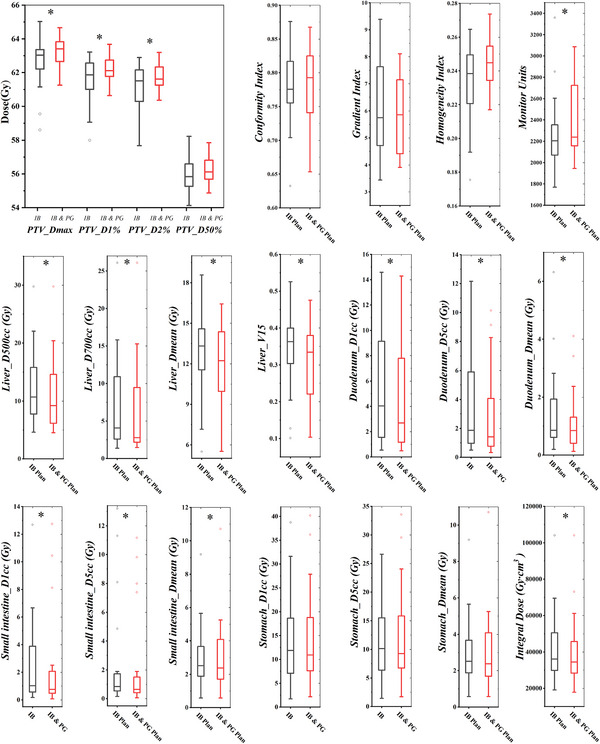
Boxplots of dose statistics to compare the IB plans with the IB & PG plans. The boxes denote median values and interquartile ranges, the whiskers indicate maximum and minimum values within 1.5 times interquartile ranges, and the dots are outliners. *Statistical significance. The IB plans indicates plans with minimum island blocking area; The IB & PG plans indicates plans with minimum combined island blocking and parked gap area.

**FIGURE 4:**
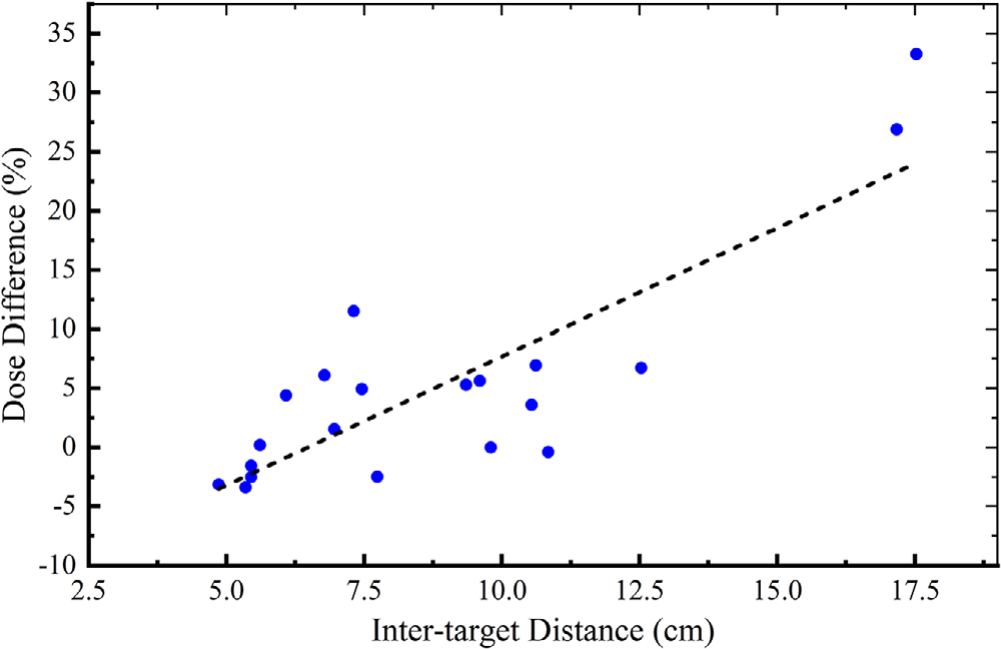
Scatterplot depicting the correlation between dose difference and inter‐target distance. Each data point represents the percentage of normal liver *D_mean_* (mean dose) sparing in IB & PG plans compared to IB plans. For patients with three targets, inter‐target distance was defined as the average distance between three target centers.

Figure 3 presents the dosimetric comparison of the IB plans with the IB & PG plans for the 20 liver SBRT cases, and Table 2 shows the percentage difference in the dosimetric metrics between IB plans and IB & PG plans. Compared to the IB plans, the IB & PG plans had higher *D_max_*, *D_1%_*, and *D_2%_* values of PTV (*P* < 0.05). The IB & PG plans had similar *CI*, *GI*, and *HI* values (*P* > 0.05) to that of the IB plans. When IB & PG optimization method was applied, the *MUs* value increased by 120.62 MU or 5.74% (median) (*P* = 0.02). In terms of OARs sparing, the IB & PG plans were superior to the IB plans. The *D_500cc_*, *D_700cc_*, *D_mean_*, and *V_15_* (*V_D_* is the percentage of volume receiving at least *D* Gy dose) of normal liver tissues in the IB & PG plans were significantly lower than those in the IB plans. The median percent reductions were approximately 5.36% (95% confidence interval (CI), 0.46% to 13.30%), 3.32% (−2.27% to 9.44%), 4.01% (−0.20% to 5.86%), 3.53% (−0.03% to 6.46%), respectively. Accordingly, in a dose level of 15 Gy, the IB & PG plans saved 14.48 cc (95% CI, −0.19 to 30.88 cc) more normal liver tissue than the IB plans. For the duodenum, the IB & PG plans showed significant improvement. The median reduction of *D_1cc_*, *D_5cc_*, *D_mean_* were 10.17%, 11.21%, and 16.03%, respectively. The IB & PG plans also significantly reduced the *D_1cc_*, *D_5cc_*, and *D_mean_* of the small intestine by 11.47%, 7.60%, and 11.25%, respectively. There were no significant dose differences for the stomach between the two plans. The *ID* was also compared in Figure 3 for all patients. The IB & PG plans provided lower (*P* = 0.008) *ID* (median, 3.45 × 10^4^ Gy•cm^3^) compared to IB plans (3.60 × 10^4^ Gy•cm^3^). The median percent dose reduction was 3.73% (95% CI, −1.89% to 8.90%). A scatterplot (Figure 4) was created to illustrate the relationship between the inter‐target distance and the percentage of normal liver *D_mean_* sparing. We found a significant correlation (*ρ* = 0.669, *P* = 0.001) between these two variants, and Figure 4 revealed a general trend toward increased sparing of normal liver tissue with increased inter‐target distance.

**TABLE 2:** Percentage difference in dosimetric metrics between IB plans and IB & PG plans.

		IB vs. IB & PG			
Metrics		Lower 95% CI	Median	Upper 95% CI	*P*‐value
PTV	*D_max_*	−1.16%	−0.59%	−0.14%	0.036
	*D_1%_*	−1.94%	−0.93%	0.21%	0.024
	*D_2%_*	−2.06%	−0.80%	0.17%	0.040
	*D_50%_*	−1.32%	−0.20%	0.14%	0.212
*CI*		−1.98%	0.47%	4.13%	0.455
*GI*		−4.08%	1.38%	6.39%	0.421
*HI*		−8.91%	−3.41%	0.10%	0.077
*MUs*		−13.19%	−5.74%	0.93%	0.020
Liver	*D_500cc_*	0.46%	5.36%	13.30%	0.024
	*D_700cc_*	−2.27%	3.32%	9.44%	0.044
	*D_mean_*	−0.20%	4.01%	5.86%	0.009
	*V_15_*	−0.03%	3.53%	6.46%	0.027
Duodenum	*D_1cc_*	3.86%	10.17%	16.51%	0.020
	*D_5cc_*	4.92%	11.21%	22.14%	0.011
	*D_mean_*	2.96%	16.03%	22.20%	0.004
Small intestine	*D_1cc_*	−0.76%	11.47%	23.89%	0.048
	*D_5cc_*	0.93%	7.60%	25.73%	0.011
	*D_mean_*	1.01%	11.25%	34.72%	0.027
Stomach	*D_1cc_*	−14.39%	−0.20%	6.19%	0.788
	*D_5cc_*	−16.26%	−1.65%	8.07%	0.681
	*D_mean_*	−5.74%	0.80%	12.13%	0.687
*ID*		−1.89%	3.73%	8.90%	0.008

*Note*: Percentage differences were calculated as (Plan__IB_—Plan__IB & PG_) / Plan__IB_.

Abbreviations: CI, confidence interval; *CI*, conformity index; *D_max_*, maximum dose; *D_mean_*, mean dose; *D_V_*, the absorbed dose that covers a specified volume; *GI*, gradient index; *HI*, homogeneity index; IB, island blocking; *ID*, integral dose; *MUs*, monitor units; PG, parked gap; PTV, planning target volume; *V_D_*, the percentage of volume receiving at least *D* Gy dose.

## 4 DISCUSSION

In this retrospective radiotherapy planning study, we developed a new collimator angle optimization algorithm based on the IB & PG area. This novel method allows us to take full advantage of collimator rotation by combining the IB and PG area. Thus, it has the potential to save more normal tissue from exposure. Two groups of test plans were generated based on the IB & PG algorithm and the IB algorithm for 20 patients with multi‐lesion liver cancer. Our result shows that the IB & PG plans reduced the dose to the liver, the duodenum, and the small intestine. The IB & PG plans also reduced the *ID* to the patient while maintaining the target coverage. To our knowledge, this is the first study that considers both the IB area and the PG area in the collimator angle optimization algorithm.

In some accelerator MLC systems, the existence of the PG problem between opposing leaves makes it necessary to rotate the collimator angle to decrease the PG area. This reduces the radiation through it. As shown in Figure 2, there are quite large differences in the calculated total area and the selected collimator angles between the IB algorithm and the IB & PG algorithm. These differences are caused by the occurrence of the PG area, which can't be shielded by the jaws in single‐isocenter multi‐lesion VMAT. In addition, the PG problem usually becomes severe when the IB problem is minimized. This is because the IB problem can be completely avoided when there is no overlap between two lesions in the Z‐direction (perpendicular to the MLC leaf movement direction). This is also the condition for the occurrence of the PG area. These findings suggest that the PG area is an important factor to consider when choosing the optimal collimator angle for multi‐lesion VMAT plans. The results also suggest that the IB & PG algorithm could reduce the total exposed area of normal tissue more than the IB algorithm could.

In this study, an analysis of the *CI*, *GI*, *HI*, and *D_V_* of PTV indicated that the IB & PG plans achieved a target dose coverage and a dose falloff beyond targets that were comparable with the IB plans. However, the *MUs* in the IB & PG plans increased by 120.62 MU or 5.74% (median) when compared with those in the IB plans. The possible explanation might be that the IB plans can achieve maximum reduction of the IB problem and simultaneously irradiate all targets to the greatest extent over the gantry arc. But the IB & PG plans also considered the PG problem in addition to the IB problem, thus parts of targets were getting blocked by the MLC when multi‐lesion shared the same MLC leaf pairs. As a result, the *MUs* of the IB & PG plans increased slightly, which caused a reduction in plan delivery efficiency. However, considering that the dose rate for the Versa HD 6 MV FFF beam is 1400 MU/min, this reduction in plan delivery efficiency is trivial.

The liver follows the parallel structure model of radiobiology and has been proven to have a strong dose‐volume effect on toxicity. Therefore, the risk of hepatic toxicity, such as RILD, is generally proportional to the mean dose and dose volume delivered to normal liver.^24^, ^25^ Pan et al.^25^ found that patients with RILD had a higher mean normal liver dose than those without RILD, and they recommended that at least 700 cc of the normal liver receive ≤15 Gy in three to five fractions. In our study, *D_500cc_*, *D_700cc_*, *D_mean_*, and *V_15_* in the IB & PG plans are significantly lower than those in the IB plans. For the IB plans, the median percent dose reductions range from 3.32% to 5.36% (Table 2). The IB & PG plans saved approximately 14.48 cc (95% CI, −0.19 to 30.88 cc) more critical liver volume in a dose level of 15 Gy than the IB plans. This result was acquired with the consideration that a *V_15_* of normal liver > 700 cc will generally be used to assess dose‐limiting toxicity, such as RILD.^26^ This result indicates that toxicity to the normal liver could be decreased by reducing the fractional volume of the normal liver tissue exposed to a threshold dose. In addition, since the IB & PG method reduced the PG area between lesions, it may help improve the high‐dose region between targets. Furthermore, we found that the dose improvement of *D_mean_* was associated with the inter‐target distance (Figure 4), which indicates that the magnitude of liver sparing was positively correlated with inter‐target distance, and the IB & PG algorithm may achieve more dose reduction in cases with greater inter‐target distance. The possible reason for this phenomenon might be that the PG area increases as the distance between targets becomes larger in most collimator angles. As a result, the IB plans have more open spaces with PG regions compared to the IB & PG plans. This causes more unwanted radiation exposure of normal liver areas. In summary, by calculating the sum of both the IB and PG regions in our optimization algorithm, the IB & PG plans save more normal liver tissue than the IB plans, which is critical in reducing the risk of developing RILD for multi‐lesion liver cancer.

Gastric, duodenal, and intestinal toxicity remain a major concern in SBRT for liver cancer. This is especially true in cases with multiple lesions. Irradiation of multiple lesions can increase the risk of toxicity because of the higher chance of multiple targets being close to the gastroduodenal loop. Multiple lesions also mean more dose spillage to OARs in single‐isocenter VMAT plans, which increases the risk of toxicity as well. Goldsmith et al.^27^ found that a *D_1cc_* of 31.4 Gy to duodenum in 3 to 5 fractions was associated with a 10% grade 2 duodenal complications risk. Lacouture et al.^28^ created a DVH risk map using published data for the small intestine. They found that a ratio of 16.2 and 21 Gy to 5 cc of small intestine in 3 fractions was associated with a 2.5% and 6.5% risk of grade 3 and higher toxicities, respectively. When comparing the IB & PG plans with the IB plans in our study, the results (Table 2) showed that the *D_1cc_*, *D_5cc_*, and *D_mean_* for the duodenum and the small intestine in the IB & PG plans were significantly reduced from 7.60% to 16.03%. Therefore, the IB & PG plans could decrease the risk of digestive tract toxicity.

Increasing dose deposition in normal tissues may play a leading role in the development of secondary cancers. Some studies showed a possible association between *ID* and secondary malignancies.^29^, ^30^ In this study, the *ID* was found to be significantly lower with the IB & PG plans (median, 3.45 × 10^4^ Gy•cm^3^) when compared to the IB plans (3.60 × 10^4^ Gy•cm^3^). This indicates that the IB & PG plans reduce the absorbed dose to the patient's body, which should decrease the secondary malignancies risk for patients receiving liver SBRT. There are several possible explanations for the reduction of *ID* in the IB & PG plans. One is that the presence of the PG problem in the IB plans increased unnecessary dose exposure to normal tissue. Another possible reason is that the reduction of the PG region in the IB & PG plans led to a smaller Y‐jaw opening since we used the jaw‐tracking function in planning. Thompson et al.^31^ measured the transmission through the Elekta Agility MLC leaf banks, and the maximum level was 0.44% at 6 MV. The transmission through the combination of leaf and sculpted diaphragm ranged from 0.08% to 0.01% at 6 MV. Huq et al.^32^ showed that Elekta MLC has higher interleaf leakage than Varian due to its distinctive MLC physical characteristics, and it requires the use of secondary collimators to reduce leakage. Therefore, the combination of the IB and PG problem in our algorithm is an effective way to decrease the *ID* in the patient's body to reduce later risk.

In most MLC systems, a minimum physical gap is required between opposing leaves to prevent possible collision of the rounded leaf ends during delivery. Several studies addressed the PG problem, and the accelerators they studied included Elekta Beam Modulator,^15^, ^33^ Varian 21EX Linear Accelerator with Millennium MLC,^34^ Elekta Agility MLC,^14^, ^35^ Varian TrueBeam Linear Accelerator,^36^ etc. The width of the PG area can vary depending on the accelerator MLC system. The parameters of the IB & PG algorithm need to be adjusted by the user based on the characteristics of the MLC and the planning system.

In our work, collimator angles were determined using different optimization methods for the two groups of plans. These collimator angles were fixed during each VMAT arc. These selected collimator angles may not appear as the optimal choice at every gantry angle since the BEVs of multiple lesions change dramatically throughout gantry rotation. Recently, some investigators^8^, ^12^, ^15^, ^37^, ^38^ proposed various dynamic collimator angle optimization methods. They found that a nonconstant collimator angle improves plan quality and time efficiency. Zhang et al.^39^ also demonstrated that collimator trajectory rotation provides an additional degree of optimization freedom for VMAT planning. However, dynamic collimator rotation during dose delivery is not yet fully available with the current linear accelerators. Thus, fixed collimator angle optimization is an effective approach that limits the unnecessary exposure of normal tissue and can speed up optimal collimator angle selection. The method used for this fixed collimator angle optimization is crucial. The optimization method must be carefully chosen to take full advantage of the potential benefits of collimator rotation. As we found in this study, the IB & PG plans provided better OARs sparing than the IB plans. In addition, it is worth mentioning that the IB & PG algorithm in our work is calculated as an equal combination of IB area and PG area. However, a carefully determined unequal combination of IB area and PG area may enhance the optimization ability of our algorithm. Several factors can affect the values of the weight coefficient of the IB and PG area, such as the magnitude of dose leakage of the PG area. An unequal combination of IB and PG area can be determined by assumption or independent optimization. The current study is an initial step towards introducing the PG problem into the collimator angle optimization process, whereas subsequent studies are ahead to improve the optimization ability of the algorithm.

Several limitations of this study should be mentioned. This is a single‐center retrospective study, and a limited number of cases were enrolled. Also, we did not have validated clinical results of different collimator angle optimization method plans. A larger cohort and a multi‐center prospective study are still necessary to further identify the dose and the clinical outcome differences between the IB & PG plans and the IB plans for multi‐lesion liver SBRT.

## 5 CONCLUSION

In this study, we presented a novel collimator angle optimization algorithm that combined IB with PG. When comparing our algorithm to the previously reported algorithm which minimized the IB area alone, the optimized collimator angles obtained from the IB & PG algorithm differed quite largely from those provided by the IB algorithm. The IB & PG plans reduced the dose to OARs and the integral dose to the patient's body. The similar dose coverage to the targets was maintained, and the normal liver sparing was positively correlated with inter‐target distance. Based on these results, we can conclude that the IB & PG algorithm can provide robust options when selecting optimal collimator angles for reducing normal tissue doses in single‐isocenter multi‐lesion liver SBRT.

## AUTHOR CONTRIBUTIONS

Conception and design: Jianghong Xiao, Maidina Abuduxiku, and Wenjun Liao. OARs and target structure delineation: Shu Zhang and Jiangping Yang. Data analysis and interpretation: Jianghong Xiao, Jiangping Yang, Xiaoqiang Chen, and Shu Zhang. Manuscript writing: Maidina Abuduxiku, Jianghong Xiao, Xiaoqiang Chen, and Wenjun Liao. Final approval of manuscript: All authors.

## CONFLICT OF INTEREST STATEMENT

The authors declare no conflicts of interest.

## Data Availability

The data that support the findings of this study are available from the corresponding author upon reasonable request.
